# Extracellular Vesicles: New Players in Lymphomas

**DOI:** 10.3390/ijms20010041

**Published:** 2018-12-21

**Authors:** Victor Navarro-Tableros, Yonathan Gomez, Giovanni Camussi, Maria Felice Brizzi

**Affiliations:** 1 2i3T Società per la gestione dell’incubatore di imprese e per il trasferimento tecnologico Scarl, University of Turin, Turin 10126, Italy; victor.navarro@2i3t.it; 2 Department of Medical Sciences, University of Turin, Turin 10126, Italy; yonathan.gomez@unito.it (Y.G.); giovanni.camussi@unito.it (G.C.)

**Keywords:** lymphoma, extracellular vesicles, immunomodulation, innovative therapies

## Abstract

Lymphomas are heterogeneous diseases, and the term includes a number of histological subtypes that are characterized by different clinical behavior and molecular phenotypes. Valuable information on the presence of lymphoma cell-derived extracellular vesicles (LCEVs) in the bloodstream of patients suffering from this hematological cancer has recently been provided. In particular, it has been reported that the number and phenotype of LCEVs can both change as the disease progresses, as well as after treatment. Moreover, the role that LCEVs play in driving tumor immune escape has been reported. This makes LCEVs potential novel clinical tools for diagnosis, disease progression, and chemoresistance. LCEVs express surface markers and convey specific molecules in accordance with their cell of origin, which can be used as targets and thus lead to the development of specific therapeutics. This may be particularly relevant since circulating LCEVs are known to save lymphoma cells from anti-cluster of differentiation (CD)20-induced complement-dependent cytotoxicity. Therefore, effort should be directed toward investigating the feasibility of using LCEVs as predictive biomarkers of disease progression and/or response to treatment that can be translated to clinical use. The use of liquid biopsies in combination with serum EV quantification and cargo analysis have been also considered as potential approaches that can be pursued in the future. Upcoming research will also focus on the identification of specific molecular targets in order to generate vaccines and/or antibodies against LCEVs. Finally, the removal of circulating LCEVs has been proposed as a simple and non-invasive treatment approach. We herein provide an overview of the role of LCEVs in lymphoma diagnosis, immune tolerance, and drug resistance. In addition, alternative protocols that utilize LCEVs as therapeutic targets are discussed.

## 1. Introduction

Lymphomas are a group of hematopoietic-derived cancers that develop from lymphocytes. The name specifically refers to the cancerous subtypes rather than to a single disease. Several lymphoma subtypes are recognized and belong to two main groups: Hodgkin lymphoma (HL), and non-Hodgkin lymphoma (NHL). HL is a rare malignancy, with an incidence of about 2.4 per 100,000 per year, and is divided into nodular lymphocyte-predominant HL (5%) and classical HL (95%). Nodular lymphocyte HL is further subdivided into nodular lymphocyte-predominant HL, nodular sclerosis classical HL, mixed cellularity classical HL, lymphocyte-rich classical HL, and lymphocyte-depleted classical HL [1]. NHL counts for 90% of all lymphomas [2]. Common NHLs affect children, adolescents, and young adults of up to 30 years of age (CAYA). NHLs in the CAYA population include lymphoblastic diseases such as acute lymphoblastic leukemia/lymphoma and ALL/LL, which originate from immature B or T cells, and Burkitt lymphoma (BL), diffuse large B-cell lymphoma (DLBCL), and anaplastic large cell lymphoma (ALCL), which originate from mature peripheral lymphocytes [3]. The most frequent NHLs are follicular lymphomas (FL) and DLBCL (70%). The histological transformation of FL to aggressive lymphoma occurs in 35% of patients and is associated with poor outcomes [4].

Six defined hallmarks allow cells to survive, proliferate, and disseminate in hematologic cancers, just as in solid tumors. These include: (1) sustained proliferative signals, (2) growth suppressor evasion, (3) replicative immortality, (4) invasion, (5) angiogenesis, and (6) resistance to cell death [5]. The acquisition of such features depends on the development of cancer cell genomic instability, immune system-driven inflammation, the reprogramming of tumor cellular energy metabolism, and tumor evasion from immune surveillance [5].

Growing amounts of evidence point to extracellular vesicles (EVs) functioning as pivotal mediators of cell–cell communication among both neighboring and distant cells in physiological and pathological settings, including hematological malignancies [6,7]. EV-specific deoxyribonucleic acids (DNA), lipids, distinct ribonucleic acids (RNA) repertoires, and proteins have been observed to change in response to different circumstances [8]. More importantly, EV cargoes profoundly differ in untransformed and transformed cells [9]. Moreover, EVs contribute to the signaling networks in the tumor microenvironment (TME) and promote tumor progression, immune escape, and chemoresistance [10]. Finally, EV cargo and their surface markers, which are parent-cell specific, are known to provide insight into tumor identity, making them valuable surrogate biomarkers [11].

Relevant data on the role of lymphoma cell-derived extracellular vesicles (LCEVs) in lymphoma diagnosis, immune escape, and chemoresistance will be discussed in this review. Moreover, an update on the exploitation of LCEVs as therapeutic targets will be provided.

## 2. EV Biogenesis

Exosomes and microvesicles are highly heterogeneous particles that originate from distinct subcellular compartments. They present a range of sizes and have diverse molecular composition. They have been classified into three main groups: (1) microvesicles (MVs), which are also referred to as ectosomes, shedding vesicles, or microparticles, and are formed by the outward budding of plasma membranes and present diameters ranging from 100 to 1000 nm; (2) exosomes, which are formed inside multivesicular bodies (MVBs) via the intraluminal budding of the endosomal membrane and are smaller than MVs, with diameters ranging from 30 to 100 nm; (3) apoptotic bodies (ABs) are large clumps of material (1000–5000 nm) that originate from cells undergoing apoptosis [12,13]. MVs and exosomes have recently been collectively defined as extracellular vesicles (EVs) [14], and this term will be used throughout this review to describe both MVs and exosomes.

EVs are important mediators of cell-cell communication that play key roles in both normal and disease processes, including cancer development and progression [15,16]. EVs contain cell-type specific cargo, including RNAs, DNA, and proteins, which are selectively sorted [17].

The mechanisms that are involved in EV biogenesis are not yet fully clear. However, it is generally accepted that the endosomal sorting complex required for transport (ESCRT)-dependent and ESCRT-independent signals play a crucial role in this process [17]. Once released, EVs induce a variety of signals to recipient cells [18]. After release, EVs are taken up by target cells. The mechanisms for EV uptake mainly include direct membrane fusion [19], ligand-receptor interactions [20], and phagocytosis [21], as well as clathrin-dependent [22] and clathrin-independent [23] endocytosis. EVs then release their cargo into recipient cells and induce a number of biological functions [24].

EV composition closely reflects their biogenesis and the environmental conditions of the cell of origin, and includes portions of cytosol and membrane-associated materials [25]. EV membranes are enriched in gangliosides and transferrin receptors [26], as well as cholesterol, ceramide, and sphingomyelin [27]. Furthermore, they contain several endosome-specific proteins, including cluster of differentiation (CD)9, CD63, CD81, tumor susceptibility 101 (Tsg101), and ALG-2-interacting protein X (Alix). EV release depends on calcium influx, cytoskeleton reorganization, and the redistribution of phospholipids in the plasma membrane [28,29].

EV composition in the physiological setting largely depends on the cell type from which they originate. They transfer information and induce the reprograming of target cells in a specific manner [30,31]. EVs transport non-coding RNA species, including RNA transcripts that overlap with protein coding regions, repeat sequences, structural RNA, transference RNA (tRNA) fragments, vault RNA, small non-coding RNA (Y RNA), and small interfering RNA, as well as other small molecules [32,33]. Besides RNAs, the presence of nucleic acids, such as mitochondrial DNA (mtDNA) [34], single-stranded DNA (ssDNA) [35], and genomic double-stranded DNA (dsDNA) [36] has been described. Although the mechanism involved in EV DNA trafficking is not clear, the transfer of packaged genomic sequences that are released into the cytoplasm after nuclear-envelope breakdown during mitosis has been postulated [37].

## 3. LCEVs and LME

**Figure 1:**
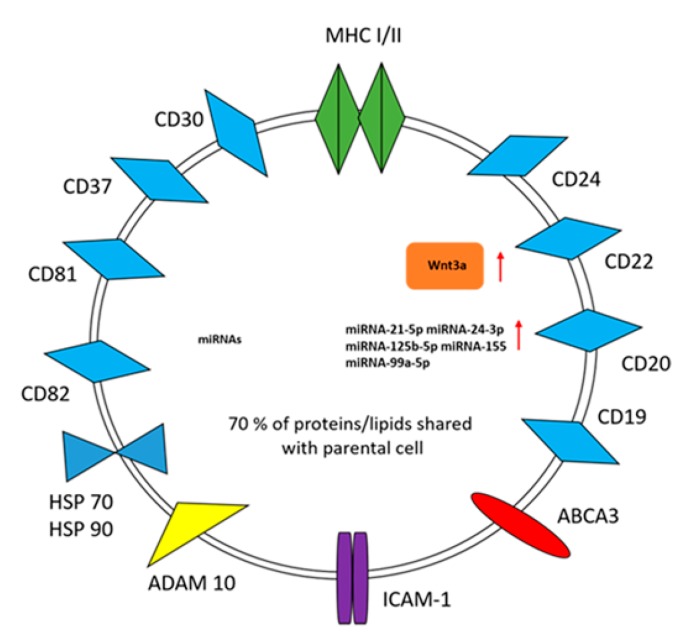
Lymphoma cell-derived extracellular vesicle (LCEV) cargo. LCEVs may contain nucleic acids, proteins, and lipids that resemble the content of their parental cell. The LCEV surface membrane is enriched with proteins that are involved in antigen presentation, signal transduction, adhesion, and drug resistance. Major histocompatibility complex (MHC) class I and II (MHC I/II); miRNA: micro-ribonucleic acid; ABCA3: ATP-binding cassette transporter A3; ICAM-1: Intercellular Adhesion Molecule 1; ADAM10: A Disintegrin and Metalloproteinase Domain-containing protein 10; HSP 70/90: Heat shock protein 70 and 90. Wnt3a: Wingless 3a. Red arrows indicate up-regulated molecules.

The molecular content of tumor-derived EVs includes a broad variety of proteins, lipids, glycans, and RNA species, and partly resembles the content of their parental cell (Figure 1) [38,39]. Furthermore, tumor-derived EVs may contain immunoinhibitory ligands and immunostimulatory molecules [40,41]. Malignant transformation is associated with increased EV release [38,42,43]. Moreover, cancer and lymphoma cells produce a large number of EVs that also contain molecules, driving the apoptosis of activated tumor-specific T cells, and thus impairing their cytolytic activity [38,39,44]. Overall, tumor-derived EVs differ from “healthy” cell-derived EVs in their functional and structural properties, as well as in their molecular profile [38,45]. Indeed, their phenotype depends on the stage and grade of malignancy [38,46]. There are increasing amounts of evidence to indicate that tumor-derived EVs preferentially target tumor cells rather than cells that are contained in normal tissues [47]. However, the precise mechanisms of interaction and affinity between tumor-derived EVs and target cells are still a matter of study.

The presence of genomic DNA inside tumor cell-derived EVs has been also reported [48]. The majority of DNA is double-stranded genomic DNA, resembling that of the cell of origin, and includes mutated and amplified oncogenes as well as transposable elements [35,36,38] (Figure 1). The function of this EV-DNA cargo is still unclear.

The importance of EVs in hematological malignancies is, in part, attributed to their role in driving the hypercoagulability state, changing the tumor microenvironment and inducing tumor evasion and chemoresistance [49].

The lymphoma microenvironment (LME) participates in lymphoma by promoting tumor cell proliferation, resistance to cell death, and evasion from growth suppressors, and provides immune escape mechanisms [4]. The LME has been increasingly recognized as a dynamic and interactive system that also supports and guides drug resistance [4]. Key factors that influence LME composition include lymphoma subtypes and signaling interactions between lymphoma cells and the cells contained within the LME. A deeper knowledge of the interaction between lymphoma cells and their non-malignant microenvironment is thought to be critical to the identification of the mechanisms involved in lymphoma initiation/development and new therapeutic targets [50].

**Figure 2:**
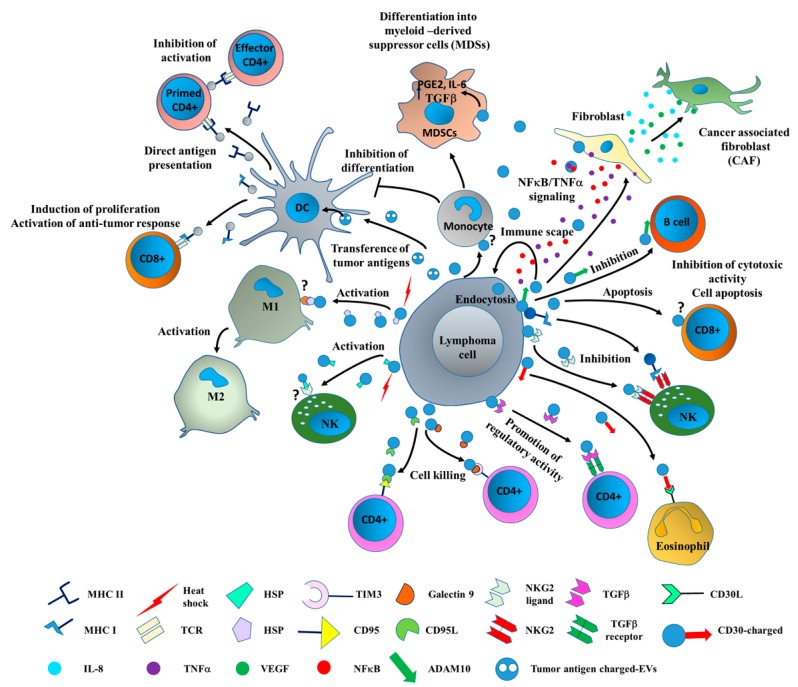
Cellular interaction in the lymphoma microenvironment (LME) driving immune escape. LCEVs contribute to tumor immune escape by coordinating the activation (black arrows) and/or inactivation (T bars) of different cells types in the tumor niche. LCEVs express different surface markers (cluster of differentiation: CD) and transport typical components resembling their cell of origin (lymphoma cell). Key molecules enriched in LCEVs interact with receptors expressed by target cells and activate intracellular signals that differentially modulate the activity of immune cells. The activation of DCs and fibroblasts further promote EV release and EV-mediated signaling events in neighboring cells. DC: Dendritic cells; NK: Natural killer cells.

The LME consists of immune cells, stromal cells, cytokines, blood vessels, and extracellular matrix components, just as the TME. Moreover, EVs have more recently been considered to be relevant LME components [39] (Figure 2). LME composition (Table 1) is dynamic and mainly guided by cancer cells. Moreover, a bidirectional flux of information between LCEVs and EVs that are derived from LME components has been reported to influence LME composition and tumor cell features [51]. Indeed, Hansen et al. [52] have demonstrated that LCEVs that are enriched in CD30 are released into the LME and take part in the recruitment of microenvironment resident cells. More recently, it has also been reported that lymphoma B cell-derived EVs that contain mutated Myeloid differentiation primary response 88 (MYD88) are involved in bone marrow LME reprogramming [53].

## 4. Immune Regulation by Tumor-Derived EVs

The immune response is made up of innate and adaptive immunity. The innate response depends on antigen-presenting cells (APCs), such as macrophages and dendritic cells (DCs), and natural killer cells (NK). The adaptive response relies on specific B and T lymphocyte activation and the generation of memory T cells. T cells are highly specialized cells that not only coordinate (T-helper: Th) and suppress (T-regulatory: Treg) the immune response, but also destroy infected cells (T-cytotoxic: CTL). Furthermore, B cells secrete antibodies that mark infected cells and pathogens in order to promote their removal from circulation [54,55].

Tumor cells, besides being characterized by unrestrained cell proliferation, possess powerful defensive mechanisms that allow them to escape immune surveillance and confer them with chemoresistance. Although EVs can display stimulatory or tolerogenic properties, depending on their packaged cargo, tumor-derived EVs mainly induce cues that collectively suppress the immune attack against tumors [56]. However, it has been shown that tumor-derived EVs are able to transfer tumor antigens to DCs by means of their major histocompatibility complex (MHC) class I expression, which, in turn, induces a CD8+ T cell-dependent antitumor immune response [57]. In order to escape immune surveillance and prepare a “niche” for newly generated and dispersed tumor cells, two main strategies are generally adopted by tumor cells: (1) the production of immune suppressive cytokines, and (2) the loss of target antigens to avoid their recognition by immune cells [58]. As a matter of fact, tumor-derived EVs deliver surface signals that modulate the expression of immunoregulatory genes, such as cyclooxygenase 2 (*COX2)*, interleukin (*IL*)-10, *CD39*, *CD73*, programmed death-ligand (*PDL)-1*, and *CD26*, which suppress regulatory T cells (Treg cells) [59]. Additionally, tumor-derived EVs can promote the production of prostaglandin E2 (PGE2), IL-6, and tumor growth factor β (TGF-β) from myeloid-derived suppressor cells (MDSC) [60]. CD8+ and CD4+ T lymphocytes are also differentially modulated by circulating tumor-derived EVs. In fact, while they inhibit CD8+ activation and proliferation, they have no effect on CD4+ T cells. Additionally, tumor-derived EVs induce apoptosis in CD8+ T cells and enhance CD4+T regulatory cell suppressor activity [61]. EV immunoregulatory effects are not limited to T and B cells, but also involve NK cells. In fact, tumor-derived EVs are known to repress NK activity via a mechanism that involves the interaction of the MHC class I-related chain (MIC) with the natural killer group 2D receptor (NKG2D) [62]. In particular, tumor-derived EVs considerably reduce the cytotoxic activity of circulating NK and generate an immunoprivileged environment that facilitates tumor escape by downregulating NKG2D [63].

Therefore, tumor-derived EVs use the above strategies to potentiate the immunosuppressive microenvironment and favor tumor growth by: (1) reprograming macrophages toward a M2 tumor-supportive phenotype [64,65]; (2) inducing cytotoxic CD8+ T cell apoptosis [64], thus lowering NK proliferation; and (3) shifting CD4+ cells to T regulatory lymphocytes [66].

## 5. EV-Mediated Lymphoma Immune Escape

Immune cells have distinct roles in lymphoma progression. They induce both immunosuppressive and antitumor immune responses. Lymphoma cells play an important role in directing tumor immune escape by creating a feasible tumor microenvironment. A number of strategies are adopted by the cells to obtain this goal: (1) the direct recruitment of immune cells [67]; (2) the indirect “education” of normal cells to release a specific pattern of cytokines or chemokines [68,69,70]; and (3) the reprogramming of resident fibroblasts to cancer-associated fibroblasts (CAF) [71]. It is known that Treg cells exert immune-suppressive activity on lymphoma-infiltrating cytotoxic T cells, resulting in ineffective immune clearance [72]. This mechanism is secondary to the recruitment, proliferation, and differentiation of naive CD4+ T cells, which is induced by chemokines and the growth factors that are secreted by the cells and/or by the LME components [4].

**Figure 3:**
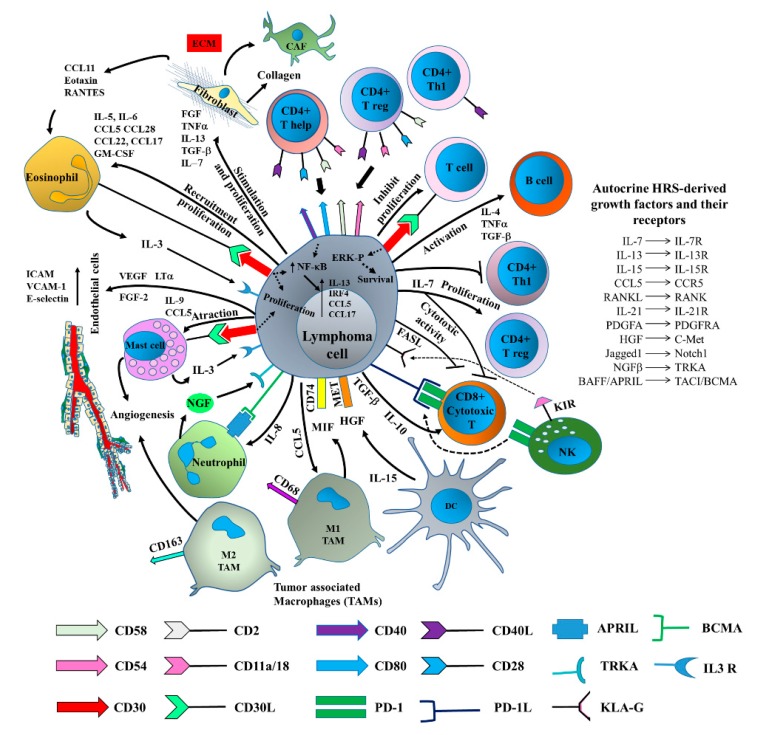
Bidirectional interaction between lymphoma cells and LME components involved in tumor immune escape. Lymphoma cells avoid the immune attack by three main mechanisms: (1) direct interaction with immune cells through surface molecules, (2) production of immune suppressive cytokines, and (3) loss of target antigens. Lymphoma cells also releases molecules that promote tumor growth and stimulate new vessel formation. Dotted lines correspond to shared receptors. Arrows indicate a direct effect. T bars indicate the inhibitory activity.

The study of tumor-derived EVs and their role in disease progression is currently one of the most attractive topics across cancer research, including that on lymphomas [39]. Despite the amount of data generated in recent years, further effort is still required if a detailed characterization of the structural and functional diversity of LCEVs and their role in immune escape mechanisms is to be achieved. LCEVs transport more than 70% of the proteins derived from their parental cells [40]. As a matter of fact, LCEVs contain discrete sets of proteins that are involved in antigen presentation, signal transduction, and cell adhesion. LCEVs also carry tumor antigens and MHC I, and are loaded with tumor surface molecules, such as CD19, CD20, and CD22, which may participate in the important signaling pathways that are involved in cell-to-cell communication in LME [40] (Figure 3). Higuchi et al. [73] have demonstrated that Epstein-Barr virus (EBV)-associated lymphomas secrete LCEVs that are mainly incorporated into monocytes/macrophages and support tumor evasion by inducing the immune regulatory phenotype in macrophages. Additionally, Ahmed et al. [74] have demonstrated that EBV infection in B cells may induce T-cell depression via LCEV-mediated apoptosis. Immune and non-immune mechanisms have been described [74]. LCEV-dependent cell-to-cell communication between distant lymphoma cells also involves educational mechanisms that are driven by CD30+ LCEVs. This mainly includes bidirectional cross-talk between malignant cells and resident fibroblasts [71]. Recent data have demonstrated that HL cells release LCEVs that express the full-length CD30 receptor and can locally modify the LME via interactions between their ligands (CD30L+) and neighboring cells. Moreover, bidirectional CD30-CD30L+ signals have been reported to contribute to the education of distant immune cells [52].

NK cells, and in particular their germline-encoded receptor NKG2D, have also been shown to play a role in NK-mediated immunosurveillance in lymphoma [75]. NKG2D is an activating molecule that recognizes self-molecules that are induced by stress [76]. Cancer cells overexpress NKG2D, and this translates into the upregulation of its ligands and more efficient NKG2D-dependent NK-mediated cell killing [77,78]. However, it has been reported that the downregulation of NKG2D-L is a common feature in LME, and is a relevant tumor-escape mechanism, as it suppresses NKG2D-dependent cytotoxicity [78]. Moreover, LCEVs that are enriched in NKG2D ligands also contribute to immune escape by decreasing the number of NKG2D-positive T cells [79].

Interference with T cell homing that prevents the systemic antitumor immune response has been associated with the overexpression of endothelin B on tumor endothelial cells in solid tumors [80]. Further studies should be performed to investigate the possibility that such a mechanism is also present in LME.

## 6. LCEVs and Angiogenesis

Increased angiogenesis has been detected in the bone marrow of patients with hematologic disorders, including lymphomas [81]. Angiogenesis is required for tumor development, growth, and invasion. The process is regulated directly and indirectly by malignant cells using a variety of pro-angiogenic and anti-angiogenic mediators, including: (1) miRNAs; (2) proteins; (3) lipids; and (4) transcription factors [82].

Tumor-derived EVs also promote angiogenesis by inducing stroma cells to release several pro-angiopoietic factors [83]. More recently, it has been shown that tumor-derived endothelial cells directly act on neighboring cells via EV-mediated miRNA transfer [84]. LCEVs that express Heat Shock Protein 70 kilodaltons (HSP-70), myelocytomatosis cancergene (c-Myc), B-cell lymphoma 2 (Bcl-2), myeloid cell leukemia sequence 1 (Mcl-1), X-linked inhibitor of apoptosis protein (xIAP), B-cell lymphoma-extra-large (Bcl-xL), and other molecules, such as phosphatidylinositol, Extracellular signal-Regulated Kinases (ERK), Mitogen-Activated Protein Kinases (MAPK), chemokines, cell surface receptors, and G proteins also act on angiogenesis [85]. Indeed, LCEVs that are released in LME actively participate in the angiogenic processes by delivering mRNA, miRNAs, and angiogenic proteins, including Vascular Endothelial Growth Factor (VEGF) [86].

## 7. Liquid Biopsy as a New EV-Based Screening Method

Solid biopsy is the gold standard for the diagnosis of a variety of diseases, particularly cancers. However, tumors that cannot be surgically treated and/or present limited accessibility are a major limitation of solid biopsies. Liquid biopsies have recently been proposed as a means to supplement surgical biopsy and resolve its limitations [87].

A “liquid biopsy” is a minimally invasive blood test that provides a consistent non-invasive clinical tool for the molecular profiling of cancer patients [87]. This approach may allow tumor cells, tumor DNA, miRNAs, and tumor-derived EVs to be identified in circulation. Moreover, liquid biopsies are currently being used to obtain more accurate diagnoses, identify specific biomarkers, and monitor disease progression, clonal evolution, and the response to treatment [88]. Accordingly, liquid biopsies have been proposed as an alternative diagnostic method for the screening of new biomarkers in LCEVs that may be translated to the clinic.

## 8. LCEVs as Markers of Disease and Progression

HL and NHL cells express more than 1000 membrane proteins, including 178 protein clusters. HL and NHL specific markers have been proposed as new molecular candidates for differential diagnosis between HL and NHLs [89]. HL cell lines and tumor tissues from HL patients express a number of proteins that are involved in immune response. Additionally, the enrichment of activated leukocyte cell adhesion molecule (ALCAM), cathepsin S, CD26, CD44, interleukin 1 receptor 2 (IL1R2), macrophage migration inhibitory factor (MIF), and thymus and activation regulated chemokine (TARC) in the plasma of HL patients has been correlated with lymphoma progression [90]. CD81+ and CD63+ LCEVs are particularly enriched in CD19, CD20, CD24, CD37, and HLA-DR [91]. The number of specific LCEVs and their surface markers also correlate with lymphoma subtypes. For example, LCEVs isolated from NHL patients are enriched in CD19 and CD20, while EVs isolated from patients with HL are enriched in CD30 [92]. In particular, CD20+ LCEVs are considered the best biomarkers for disease progression and antibody-based treatment response, as their circulating level directly correlates to the CD20+ circulating cells in patients [93]. Sera from HL patients also contain a high number of CD30+ LCEVs [92], which has been correlated with patient outcomes [94]. Therefore, CD30+ LCEVs, besides representing a valuable diagnostic marker, might also be used to monitor responses to treatment [95]. Likewise, Tosetti et al. [96] have demonstrated that LCEVs released by HL cells are enriched in the mature bioactive form of ADAM10. In addition, Jones et al. [97] have demonstrated that miRNA-21 and miRNA-155 are enriched in the sera of HL patients. Accordingly, a standardized size-exclusion chromatography (SEC) analysis has demonstrated the presence of more than 400 miRNAs in the highly enriched populations of HL patient-derived LCEVs. miRNA-21, miRNA-155, miRNA-21-5p, miRNA-24-3p, miRNA-127-3p, and let7a-5 are found among them [97]. A large Spanish multicentric study has recently demonstrated the feasibility of monitoring cancer evolution by measuring B-cell lymphoma 6 (*BCL-6*) and *C-MYC* mRNA levels in LCEVs isolated from the plasma of patients with B-cell lymphomas. The study also demonstrated that both markers are predictors of worse progression-free survival (PFS). Moreover, it has been shown that LCEV C-MYC mRNA content may also predict poor prognosis and/or incomplete treatment response [98]. The relevance of LCEVs as potential predictors of drug efficacy has also been demonstrated in patients with diffuse DLBCL. The observation that two miRNAs (miRNA-99a-5p and miRNA-125b-5p) were enriched in DLBCL EVs was found to correlate with shorter progression-free survival and drug resistance [99]. Finally, a direct correlation between circulating LCEV number, disease progression, and response to treatment has been reported [38]. These observations support the notion that LCEVs can be considered not only as potential markers of disease and disease progression, but also as biomarkers to monitor response to treatment.

## 9. LCEVs as Mediators of Drug Resistance

The identification of a suitable B-cell target antigen, CD20, in the early 1980s paved the way for the development of monoclonal antibody technology, and in particular promoted an immunological therapeutic approach to NHL patient treatment [100]. CD20 is a membrane-spanning phosphoprotein that is expressed by late pre-B and mature B cells, as well as by the majority of NHL B cells. The absence of this marker on early B-cell progenitors and immature cells facilitated the development of a specific antibody-based therapy that can recognize human CD20 and induce complement-dependent (CDC) and antibody-dependent cellular cytotoxicity (ADCC) [101]. Rituximab was the first anti-cancer biological drug to be approved as an anti-CD20 antibody (RTX; Rituxan^®^, MabThera^®^) by the United States (US) Food and Drug Administration in 1997 [102]. 

The last decade has seen a new anti-CD20-based therapy for lymphoma treatment being developed and approved [103]. Unfortunately, it is becoming evident that B-cell lymphoma cells release CD20+ LCEVs. It has been suggested that circulating CD20+ LCEVs capture rituximab, and thus hamper its therapeutic effect. This seems to be particularly relevant at the beginning of treatment. In particular, it has been demonstrated that the high number of circulating CD20+ LCEVs can “sequestrate” rituximab, reducing the effective number of deposable molecules, and in turn, reducing its therapeutic effectiveness [104]. Moreover, it has been reported that LCEVs efficiently extrude drugs and can drive drug resistance in aggressive B-cell lymphomas via the ATP-transporter A3-mediated mechanism (ABCA3) [105]. The expression of HSP-70, c-Myc, Bcl-2, Mcl-1, xIAP, and Bcl-xL and other molecules, such as phosphatidylinositol, ERK, MAPK, chemokines, cell surface receptors, and G proteins in LCEVs has also been associated with resistance against humoral immunotherapy [85]. Moreover, the increase in ADAM10 activity that is mediated by LCEVs has been reported to interfere with immunotherapeutic approaches. The release of Tumor Necrosis Factor (TNF)α, soluble MHC I polypeptide-related sequence A (sMICA), and soluble CD30 (sCD30) has been reported as a crucial mechanism [96]. This observation has led to specific ADAM10 blockers being proposed to boost the anti-lymphoma immune response and/or drive efficient antibody-drug-conjugate (ADC)-based and Ab immunotherapy [96].

## 10. EV-Based Lymphoma Therapies

**Figure 4:**
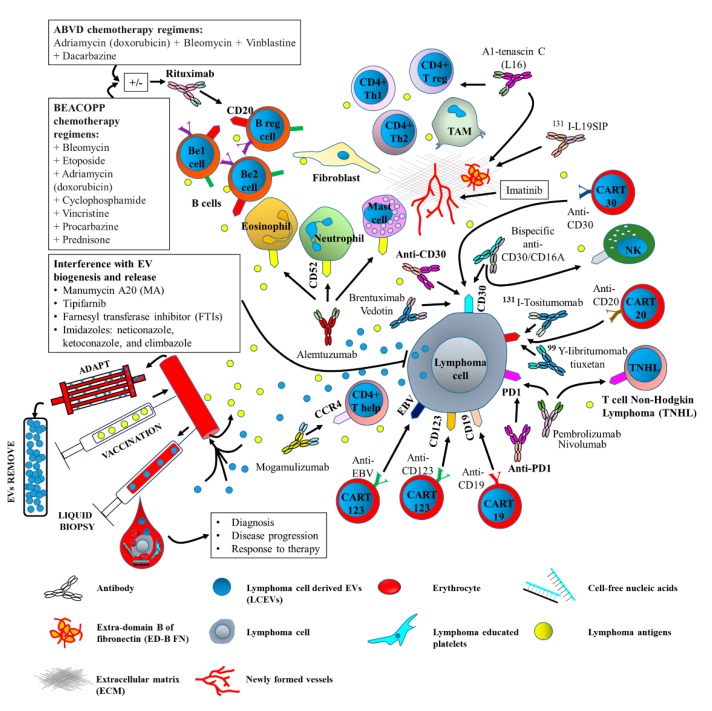
Scheme of therapeutic approaches (currently used or under investigation) and liquid biopsy using LCEVs. Oncogenic drugs and specific antibodies targeting surface molecules expressed by LME components represent the main therapeutic approaches used in clinic. Current clinical trials are also evaluating new potential approaches using vaccinations with tumor antigens. ADAPT represents a potential approach to remove LCEVs from the bloodstream. Liquid biopsy has been proposed as screening method for diagnosis, disease progress, and response to therapy. New potential approaches are represented by the chimeric antigen receptor (CAR), which is introduced in immune cells to retarget their cytotoxicity toward specific tumor antigens (CART). CARs are synthetic proteins generated by the fusion of a single chain variable fragment (scFv) derived from a monoclonal antibody with the signaling and costimulatory machinery of the T-cell receptor (TCR) [107]. CART cells have been evaluated as promising new cell-based therapy approach using CD19 [108], CD30, CD123 [109], and the Epstein-Barr virus protein (EBV) as molecular targets [110]. ADAPT: Adaptive dynamic artificial poly-ligand targeting.

Tumor-derived EVs are involved in cancer development on multiple levels. It is worth noting that EVs also drive “adaptation/defense” and chemoresistance mechanisms [106]. This has spurred current research toward feasible approaches that can interfere with the formation/release or spread of tumor-derived EVs. Researchers are currently focusing on four main areas: (1) EV-based vaccines; (2) the development of specific antibodies to neutralize tumor-derived EVs; (3) the targeting of EV biogenesis; and (4) the extracorporeal removal of tumor-derived EVs (Figure 4).

### 10.1. EV-Based Vaccines

Cancer vaccines are considered a class of therapeutics and are also called biologic response modifiers (BRM) [111]. Single or combined tumor antigens are injected to drive immunoprotection [112]. The identification of selective antigens that are abundantly expressed on cancer cells has set the basis for the design of a number of cancer vaccine trials [113]. However, there are few data to demonstrate the feasibility of monitoring the immune response, meaning that vaccination is not yet considered a feasible therapeutic approach for lymphoma [114].

The heterogeneity of the molecular profile of tumor cells during progression and after treatment is considered the most relevant limitation of vaccination-based cancer therapy. Moreover, antigen-specific T-cell repertoire diversities, and the presence or absence of pre-existing immune tolerance, have also contributed to unsuccessful responses to vaccine-based therapy [115].

It has been reported that HL-derived LCEVs are able to induce a potent and specific cytotoxic response in CD8+ T lymphocytes. This effect relies on the upregulation of MHC molecules, costimulatory molecules, and cytokines by LCEV-mediated DC functional maturation [85]. DCs, which are the most proficient APCs, can take up tumor antigens and, depending on environmental cues, present antigens at tumor sites and lymphoid organs to either prime, sustain, or abrogate EV-based active vaccination [116]. Menay et al. [117] have demonstrated that T-cell lymphoma-derived LCEVs are able to induce an efficient immune response and memory against lymphoma. This effect was associated with T helper 1-mediated responses. However, Chen et al. [85] have more recently demonstrated the presence of a dual LCEV-mediated effect. They showed that LCEVs may display immunosuppressive activity that can promote in vivo tumor growth. On the other hand, they also demonstrated that T cells, which were recovered from tumor-bearing mice treated with LCEVs, displayed increased anti-lymphoma activity. This effect relies on DC-mediated T-cell expansion and the inhibition of Th2 immunosuppressive activity [85]. These data suggest that the education of DCs using LCEVs as tumor-associated antigens may be a novel DC-based immunotherapy [85].

More recently, the combined use of standard-of-care chemotherapeutics and EV-based vaccines has been proposed as a means to modulate immunity in solid tumors. For example, cyclophosphamide has been shown to amplify the cytotoxic T-cell response against cancers when combined with DC-derived EVs [118,119]. Future effort should be devoted to an evaluation of the effectiveness of this combined approach as an alternative treatment in lymphomas as well.

### 10.2. Neutralizing EV-Based Approaches

Although rituximab has been shown to improve the overall survival of patients with both aggressive and indolent B-cell NHL [120], remission is only achieved in 50% of CD20-positive lymphoma patients [120]. This indicates that additional and more specific antibodies are required. CD19, CD22, CD37, and CD40 have been identified, thanks to lymphoma molecular profiling [89], and are currently under investigation for the development of new antibody-based treatments. A number of new specific antibodies, including the anti-CD22 [121], anti-CD40 [122], anti-CD19 [123], anti-CD19/CD3 [124], and anti-CD37 antibodies [125], are currently used for alternative antibody-based therapy in lymphomas. Since these markers [40] are also expressed by LCEVs, these approaches, if validated, could also be used to remove LCEVs from circulation. Therefore, a dual effect may be sought.

### 10.3. Targeting EV Biogenesis

The blockade of EV release from tumor cells has been proposed as an alternative EV-mediated therapeutic approach in cancer [126]. The identification of key cellular pathways that are involved in EV biogenesis would be crucial to this purpose. It has been demonstrated that EBV infection modulates EV biogenesis and the miRNA content in Burkit lymphoma cells [127]. However, the complex biological process of EV trafficking and the large number of proteins involved represent a significant challenge.

Despite the enormous amount of effort that has been made to identify the signaling pathways involved in LCEV formation and release, few data are currently available. Koch et al. [105] have recently shown that LCEV biogenesis is strictly dependent on ATP Binding Cassette Subfamily A Member 3 (ABCA3) expression, and that genetic ablation and chemical interference with ABCA3 expression improve drug accumulation in the nucleus of B-cell lymphomas.

The inhibition of sphingolipid ceramide formation via a range of approaches has also been shown to interfere with intra-endosomal membrane transport and EV generation [17,128]. Several imidazoles (neticonazole, ketoconazole, and climbazole) as well as manumycin A20 (MA) and tipifarnib, which are two farnesyl transferase inhibitors (FTIs), have been found to inhibit the specific pathways that are involved in EV biogenesis in cancer cells [129].

The Rab family is known to control vesicular trafficking, including its motility, docking, budding, and the fusion of numerous vesicular transport intermediates [130]. More importantly, the enrichment of Rab35 on EVs has been shown to play a crucial role in the regulation of vesicle density [131]. Ostrowsky et al. [132] have demonstrated that several Rab family members are also involved in EV biogenesis and provide noticeable results, which strengthens the concept of using Rab27 inhibitors to interfere with EV biogenesis.

The role of intracellular Ca^2+^ concentration has also been reported to contribute to EV biogenesis in erythroleukemia cell lines. This was observed using dimethyl amiloride (DMA), which is an inhibitor of Na^+^/H^+^ and Na^+^/Ca^2+^ exchangers. In particular, it has been shown that DMA is able to decrease constitutive and Ca^2+^-dependent EV release [133]. DMA was also found to reduce the secretion of LCEVs in mice that bear lymphomas. This translates into the inhibition of myeloid-cell suppressor functions and an improvement in immune surveillance [134].

### 10.4. Extracorporeal Removal of LCEVs

EV dialysis has been proposed as a novel approach for the removal of EVs in cancer and lymphoma patients [135]. However, no solid data on EV removal by hemofiltration are yet available. Nevertheless, Aethlon Medical has proposed a therapeutic hemofiltration approach, termed the Aethlon ADAPTTM (adaptive dialysis-like affinity platform technology) system, which consists of immobilized affinity agents in the outer-capillary space of hollow-fiber plasma separator cartridges integrated with either a standard dialysis unit or with devices approved for continuous renal replacement therapy (CRRT) [135]. So far, the combination of this technology with dialysis has been applied to virus reduction/clearance in hepatitis C-infected patients. Feasibility was indirectly supported by a reduction in the number of infected cells [135]. Therefore, its potential application in LCEV removal still requires validation, despite this approach being a valuable innovative option.

## 11. Conclusions

EVs have been increasingly considered simple and accurate non-invasive biomarkers in many cancers [11]. In particular, LCEVs have been investigated as novel “multiomic shells” for disease detection, immune escape, response to treatment, and drug resistance. Moreover, the cross-talk that occurs between malignant cells, immune cells, and other LME components via LCEVs has gained particular attention for its role as a driver of lymphoma progression and treatment response [6]. Recently, it has been reported that circulating EVs that bear programmed death-ligand 1 (PD-L1) change during treatment, due to specific immune responses [136]. This suggests that LCEVs could be used as real-time biomarkers for treatment response [137]. Moreover, the use of LCEVs as biomarkers of treatment response would be particularly relevant for patient management during treatment, as the International Prognostic Index (IPI) predicts survival time, but not treatment efficacy [138]. Furthermore, most lymphoma-associated antigens being expressed on LCEVs has led to the proposal of the possibility of using them for immune approaches [38]. This is further supported by the finding that LCEVs carry a large number of proteins that can be considered potential target candidates [40]. Effort has also been focused on the identification of the mechanisms that regulate LCEV biogenesis. This would allow the development of novel therapeutic approaches in which the targeting of LCEVs might interfere with tumor growth. This would also be particularly relevant if interfering agents could be directed to CD20+ LCEVs, which are known to act as decoy targets to facilitate tumor escape from humoral immunotherapy [139]. However, a refined and detailed understanding of LCEV actions is still required if advanced therapeutic options are to be successfully developed. Moreover, an in-depth investigation of the relationship between LCEVs, disease aggressiveness, and recurrence would also provide assistance. These topics must be addressed before the LCEV field can be translated to the clinic.

## Figures and Tables

**Table 1:** Lymphoma Microenvironment (LME) composition.

LME-Associated Composition	Cell Types
Immune cells	Cytotoxic T cells (CTLs)
	Follicular B helper T cells (TFH)
	Regulatory T cells (Treg)
	Natural Killer cells (NK)
	Bystander B cells
Stromal cells	Mesenchymal stromal cells (MSCs)
	Lymphoma associated macrophages (LAMs)
	Myeloid-derived suppressor cells (MDSCs)
	Dendritic cells
Extracellular components	Extracellular matrix (ECM)
	Cytokines/Chemokines
	Lymphoma exosome
